# Supplementary material for: Are carbon emissions trading and green financial instruments synergistic? -Comprehensive quantitative research based on content analysis
Source: PLoS One. 2024 Mar 7;19(3):e0298601. doi: 10.1371/journal.pone.0298601 (PMC10919601; doi:10.1371/journal.pone.0298601)
Supplement: S1 Data — (ZIP) [file pone.0298601.s001.zip › Data/地级市Z_F工作报告.zip!/║┌┴·╜¡/╝╤─╛╦╣╩╨/└┤╫╘╣1⁄2╓┌║┼í╛┬φ┐╦╩2╛▌═°í┐_2016.docx]

各位代表：

现在，我代表市人民政府向大会报告工作，请予审议，并请各位政协委员提出意见。

一、2015年工作及“十二五”发展回顾

过去的一年，面对严峻的压力和挑战，我们在省委、省政府和市委的坚强领导下，团结带领全市人民，坚持稳中求进工作总基调，以提高经济发展的质量和效益为中心，注重创新驱动，狠抓改革攻坚，积极改善民生，推进依法行政，在困难超出预想的情况下，工作成果好于预期，主要指标增速位居全省前列。预计全年地区生产总值实现820亿元，增长6.5%。固定资产投资完成533.9亿元，增长10.5%，位居全省第一位；社会消费品零售总额实现401亿元，增长10.5%，位居全省第三位；一般公共预算收入36亿元，超出预算1.4个百分点；城镇居民、农民人均可支配收入超过经济发展增速。

（一）项目建设扎实推进。持续开展项目攻坚，实施基础设施项目299项，完成投资164亿元，哈佳高铁、同江中俄铁路大桥及水利工程等重大项目进展顺利。入库PPP项目16个，总投资123.5亿元。全市开复工千万元以上产业项目709项，完成投资369.7亿元，占固定资产投资的69.2%；竣工项目532项。26项省重点推进产业项目全部开复工建设，完成投资计划的112%。谋划生成产业关联度高、符合市场规律、具有可持续市场赢利模式项目312项。创新招商模式，引进亿元以上项目136个，甄别筛选有效项目线索320个，国内招商引资到位资金564亿元，增长30.5%，实际利用外资2.43亿美元，增长5%。通用航空基地等项目实现签约，百威英博年产100万千升啤酒一期等项目投产运行，泉林秸秆综合利用一期项目部分竣工投产，胡商国际佳大商业广场等项目开工建设。

（二）农业转型步伐加快。深化两大平原综合配套改革。创新农业经营主体。全市新增农民专业合作社483个、家庭农场205个、专业大户41个，土地流转面积811.8万亩；规模以上龙头企业引领带动29万农户参与产业化经营。创新社会化服务体系。开展科技人员包保责任制试点，全市产加销一体化服务合作社达到395个，桦川新峰、汤原维维汤旺河等近百家工商企业参与社会化服务。创新农村金融，涉农贷款增长15%，利率下降2个百分点。创新农产品销售体系。组建时代农业公司，建立佳木斯市大米标准体系，重点打造地理标识品牌；推进“互联网+农业”、农超对接等流通业态，50余家企业与合作社实现线上销售。加快绿色特色农业发展。新增国家级绿色食品原料标准化生产基地认证面积130万亩，同江市绿色食品质量安全监管实现全覆盖；以汤原亮子奔腾等企业为依托，带动食用菌栽培形成新的产业模式和产业规模，紫苏、红树莓等特色种植面积不断扩大；建成“两牛一猪”标准化规模养殖场54个；“桦南紫苏”国家地理标识申报成功。全市建成“一村一品”专业村95个。富锦幸福灌区实施委托运营，农田水利设施市场化改革取得实质性进展。全市粮食总产实现十二连增。

（三）工业经济企稳运行。推进服务常态化，出台市级惠工政策3项，帮助企业解决实际困难。及时兑现国省优惠政策，争取各类扶持资金1.3亿元，落实企业贷款22亿元，减免各类企业和经营实体税金2.35亿元，组织企业开展国际国内对接推介。中国建材集团与佳星玻璃战略重组基本完成，七彩毯业、三和亚麻等企业生产经营趋于稳定。全市新增小微企业2676户。新建产业技术创新联盟、科技企业孵化器各3家，新增国家高新技术企业3户，科技成果转化签约额超过5000万元，实现高新技术产值203亿元。

（四）服务业发展向快向好。传统商贸转型升级。万达商业广场等大型商贸项目建成投入运营，全市新增大型商业网点营业面积26万平方米；与京东等国内知名大型电商合作，大商集团等传统商业实现线上线下运营。文化旅游融合发展。投资1.1亿元，推进7个旅游项目建设，晋升国家4A级、3A级旅游景区各1个，街津口赫哲族民俗体验区，被农业部认定为全国休闲农业与乡村旅游示范点，全市旅游收入18.7亿元，增长16%。养老产业扎实起步。与15家省外养老机构签订意向候鸟式养老协议。金融支撑实力增强。广发等3家银行入驻我市，金融机构贷款余额增长53%。适时出台扶持政策，促进房地产市场平稳发展。

（五）改革开放取得进展。推动关键领域改革。实行“多取消、审一次、真备案”，深化行政审批制度改革，建立权力清单、责任清单，在全国率先推行行政职权运行流程标准化管理，优化331项行政职权审批流程，签批环节、审批时限分别减少52%、66%；市直及中省直行政职权精减46.4%；累计向城区放权587项，下放编制652个。推动公共资源市场化配置改革，市公共资源交易中心投入使用，政府投资建设工程交易项目190项，政府集中采购115笔，采购成本降低9.9%。探索财政预算绩效管理改革，市本级预算、“三公”经费预算和部门预算全部公开，30万元以上项目资金实行绩效目标管理，“三公”经费减少11.3%。清理非办公类土地资产面积77.5万平方米、房屋资产面积4.3万平方米。深化事业单位改革，整合撤销5家单位。启动社会诚信代码改革，开展综合执法体制改革。厂办大集体改制基本完成。进一步提高对外开放水平。对接“一带一路”和“龙江丝路带”战略规划，争取佳木斯宏力港等重大项目24个。中俄同江和下列超前发展园区规划建设进展顺利，全市在俄年度投资增长13%。同江经济开发区被列为省重点对俄进出口加工园区。建成佳天国际农副产品物流交易中心海关监管库。成功举办中俄（佳木斯）农机境外展，组织参加16个境内外大型展会，达成合作意向69个。我市至哈巴、首尔航线稳定运行，机场客运量增长6.1%。开展与俄、韩等国互访活动94次，对外人文交流与经贸合作日益密切。受国际宏观经济形势影响，贸易额有所下降，但进出口贸易对本市经济的拉动作用有所提高，进口产品落地加工比重提高7.35%，出口产品本地化率提高8.27%。

（六）城乡环境更加宜居。加强城市路网建设，新建续建城市主干路11条、高新区道路4条，维修改造主次干道48条、区街巷道56条、桥梁11座，群众出行更加便利。实施公路交通安全隐患整治，改造平交道口2327个、危桥90座。推进9项专项整治，健全城市管理监督机制，郊区、向阳区开展“绿色清雪”试点，城市管理得到加强。新建长安公园，对8个公园、102条街道进行维护改造和绿化提档升级。取得国家智慧城市试点资格，完成海绵城市试点申报工作。松花江水污染防治规划项目建成率居全省首位，松花江佳木斯断面水质达标率100%。落实空气质量监测新标准，良好以上天数达到338天。沿江、沿河工业排污口全部达标排放。生态创建工作位于全省前列。造林9.27万亩，超额完成省定任务。推广郊区模式，实施205个村的美丽乡村建设，农民参与建设的积极性更加高涨，得到省委、省政府的充分肯定。

（七）民生事业彰显实效。贴近群众需求办好民生实事，全市民生投入140亿元，年初确定的任务全部完成。城镇新增就业7.2万人（次）；创建全省首家大学生创业社区；发放小额担保贷款1.8亿元，城镇登记失业率4.05%。城乡“五险”继续扩面，保障水平得到提高，率先在全省实现城镇基本医疗保险市级统筹；启动城镇居民大病保险；社保卡发放60万张。城乡低保、五保供养、市区最低工资全部达到省定标准。争取政策性贷款40亿元，实施棚户区改造104万平方米，新增廉租补贴1039户；完成农村泥草房改造10771户。公积金归集额、贷款额分别增长13.9%、178.1%。解决200个村屯、15.4万人的饮水安全问题。6.6万农村贫困人口实现脱贫。市区节能改造151.8万平方米，改造老旧小区楼房719栋；解决居民商品住房不能办照28416户；新建改建公厕22座；新增停车泊位2489个；公共自行车服务系统投入运行；更新大容量新型环保公交车35台，新增、调整公交线路11条；改造供热管网32.9公里，淘汰小锅炉89个，供热质量明显改善；新建改造燃气管网62公里，新增居民用户1万户。义务教育阶段全部实行“阳光招生”、“阳光分班”。推进3个省级临床重点专科和8个特色品牌专科建设，医疗质量稳步提高；推行医疗服务“一卡通”，实行专家“全日制”门诊，群众满意率明显提高。市级“三馆”标准提升，县级“两馆”提档升级。完成165个中心村文化广场、文化活动室建设任务；县城数字影院实现全覆盖。市区社区居委会组织机构成员工资及待遇纳入市级财政预算，收入水平得到提高；新增省级“双百”示范社区17个；大型商业区、快捷宾馆、餐饮消费场所等均已开通无线WIFI。佳木斯快乐舞步获“2015年全国广场舞操大赛决赛”一等奖。立体化社会治安防控体系初步形成，打防管控能力明显增强，刑事案件发案率下降、破案率提高，社会治安形势持续向好。安全生产事故及控制指标明显好于年初预估，化解重点信访案件286件，社会保持和谐稳定。

（八）政府建设得到加强。依法健全和完善政府履职决策机制，增设专家咨询机构，建立法律顾问制度，政府运行更加规范。推进政务公开，自觉接受监督，100%办复人大代表建议和政协提案；畅通民意维权渠道，全市受理行政复议案件数量增加近六倍；推行网上问政，在政民互动中咨询答疑，解决实际问题，服务指向更加准确。深入开展“三严三实”专题教育活动，对梳理出的43个问题，落实责任，发力整改，行政效率不断提高，工作作风更加务实。加强行政监督、审计监督，推进廉政建设，查处各类违纪案件827件，发展环境有所改善。

一年来，市场监管、外事侨务、民族宗教、机关事务、统计、气象、地震、人防等工作得到加强，妇女儿童、残联、红十字等工作扎实推进。

在总结成绩的同时，我们也必须正视当前发展面临的突出问题，主要体现在：传统工业整体实力不强，缺乏核心竞争力，创新能力不足，产销下行压力较大；农业大而不强，农民持续增收的难度加大；财政收入结构不尽合理，财力不足，政府偿债和重大工程建设资金配套集中，保障发展的财政资金收支矛盾较大；改革工作进展不平衡，一些攻坚任务没有取得突破性进展；民生领域部分遗留问题尚未妥善解决，影响社会和谐稳定的矛盾依然存在；政府部门少数人员责任意识和专业水平不强，行政理念更新与实际工作需要存在差距等。对此，我们将高度重视，并在今后的工作中认真加以解决。

各位代表，刚刚过去的2015年，是实施“十二五”规划的收官之年。五年来，我们逐步认识新常态，落实新要求，运用新的思维理念，破解发展难题，激发担当创新精神，推进工作落实，取得了经济社会发展的积极成效。2015年地区生产总值、固定资产投资、社会消费品零售总额、城镇居民人均可支配收入、农村居民人均可支配收入分别是2010年的1.6、2.4、1.9、1.9、1.8倍。主要污染物减排提前一年完成“十二五”目标。

五年的实践步履坚实，取得的经验弥足珍贵。一是坚持勇毅笃行，保持定力。遵循科学发展思路，系统谋划发展格局，实而不浮搞建设，驰而不息抓产业，力求在“好”的前提下能快则快，综合竞争实力不断提升，为加快发展奠定了坚实基础。二是注重抢抓机遇，奋发有为。认真研判形势，找准上级政策与我市发展的契合点，围绕“四大优势”持续精准发力，开工建设一批补短板、增后劲的重大项目，为加快发展打造了重要引擎。三是推进改革创新，激发活力。坚持市场化导向、法治化原则、优质化服务，努力构建有利于科学发展的体制机制，内挖潜力，外聚要素，为加快发展拓展了空间。四是围绕需求导向，惠民利民。注重民生工作的持续性、渐进性、积累性，立足当前办实事，着眼长远建体系，让群众真正得益受惠，为加快发展营造了和谐稳定的社会环境。所有这些，是过去五年我市取得工作成效的重要经验，也是未来实现发展目标的宝贵财富。

各位代表，五年来政府工作成绩的取得，是省委、省政府和市委正确领导的结果，是全市上下共同努力、社会各界鼎力支持的结果，是历届政府一步一个脚印打下坚实基础的结果。在此，我代表市人民政府，向全市广大干部群众，向各民主党派、工商联、人民团体、离退休老同志，向驻佳部队和武警官兵，向各中省直单位，向所有关心支持佳木斯发展的海内外朋友们，致以崇高的敬意和衷心的感谢！

二、“十三五”期间的目标任务

今后五年，是我市全面建成小康社会的决战决胜阶段，也是实现更大突破的重要战略机遇期。综合研判形势，我国经济发展长期向好的趋势没有改变，国家推进“一带一路”、全面振兴东北等老工业基地、“互联网+”、《中国制造2025》、脱贫攻坚，黑龙江“五大规划”、“龙江丝路带”、十大重点产业等战略措施深入推进，我市区域中心城市、现代农业、沿边开放等优势潜力不断释放，哈佳高铁、同江中俄铁路大桥、三江连通工程等重大基础设施建设步伐加快，象屿粮食深加工、泉林秸秆综合利用等一大批重大产业项目加速推进，新的增长因素和力量不断汇集，将为佳木斯发展创造更好条件。同时，我们也深刻认识到，在当前经济总体增速放缓的新常态下，诸多矛盾和风险隐患的挑战依然严峻，加之自身创新能力较弱，经济总量规模小，新的发展动能尚未完全形成，也对我市结构调整提出了更新更高的要求。

根据市委关于制定国民经济和社会发展第十三个五年规划的建议，“十三五”时期全市经济社会发展的指导思想是：全面贯彻党的十八大和十八届三中、四中、五中全会，特别是习近平总书记系列重要讲话精神，全面落实“四个全面”战略布局，自觉践行五大发展理念，创新实施省委“五大规划”战略部署，深入推进“龙江丝路带”建设，结合我市发展的阶段性特征，以加快发展为中心，突出动能转换，着力推进“七大战略任务”，进一步提高发展质量和效益，进一步改善民生事业，进一步促进社会公平正义，进一步创新社会治理和政府服务，全面建成发展有质量、百姓得实惠、生态环境好、社会更文明的小康社会。

——综合实力明显增强。坚定不移稳增长，实现经济和社会发展规模与质量、速度与效益、增长与转型的协调统一，到2020年，全市地区生产总值和城乡居民人均收入比2010年翻一番，地区生产总值年均增长6.5%以上。

——创新动能更加有力。科技、管理、品牌、组织、商业模式等方面的创新工作统筹推进，原始创新、集成创新和引进消化吸收再创新能力得到提升，推动一二三产融合发展，产业结构优化升级，在新一轮经济发展变化中打造区域竞争新优势。

——重点改革实现突破。行政体制改革不断深化，政府效率和效能持续提高。供给侧结构性改革取得实质性进展。支持非公经济发展的政策体系和促进机制更加健全，发展环境有实质性改善。“两大平原”综合配套改革试验阶段性任务全面完成。

——绿色理念深入人心。构建绿色、低碳、循环发展的产业体系，建设循环经济示范城市和再生资源试点城市。强化生态文明建设的制度保证，绿色发展方式和生活方式逐步形成，绿色引领时尚，保持最美生态。

——城市功能日益完善。集中开展高铁、公路、水利等重大基础设施攻坚，完善相关配套设施，强化“五个区域中心”建设，提升城市综合竞争实力和辐射吸引力，打造宜居、宜业、宜商、宜游的黑龙江省东部城市群中心城市。

——开放拉动能力增强。同江中俄铁路大桥项目取得重大进展，跨境合作通道优势全面提升。“龙江丝路带”重要枢纽和窗口作用更加突出，开放发展要素更多集聚，外向经济功能更加齐全，对外开放空间不断拓展，对外交流合作深化升级，构筑沿边开放新高地。

——发展成果全民共享。新型城镇化和美丽乡村建设有序推进，城乡发展更加协调。就业、教育、文化、社保、医疗、住房等公共服务体系更加健全。坚决打赢脱贫攻坚战，全面建成小康社会，让全市人民享有更多获得感、幸福感。

三、2016年工作安排

今年政府工作的总体要求是：以全面建成小康社会为目标，深入贯彻落实中央和省委的决策部署，按照市委十二届六次全体会议的要求，围绕市场导向，在产业创新扩量、农业提质增效、非公经济和县域经济发展、企业降成本增效益等方面持续发力，进一步深化体制机制创新，推动多角度开放合作，加快基础设施建设，切实保障改善民生，维护社会和谐稳定，促进经济社会发展既有量的扩张，更有质的提升，实现“十三五”规划的良好开局。

综合考虑各种因素，主要预期目标是：地区生产总值增长6.5%左右，固定资产投资增长10%左右，社会消费品零售总额增长8%左右，城乡居民收入增长与经济增长同步。

（一）强化项目建设，扩大有效投资。把上项目扩增量作为一号工程，提升项目建设的规模和质量。

抓好基础设施建设。加快哈佳高铁项目建设，争取牡佳客专项目开工，推动佳木斯站综合改造、松花江二桥、悦来航电枢纽等重大项目取得积极进展。开展产业项目攻坚。开复工产业项目500项，完成投资400亿元以上。重点推进泉林秸秆综合利用二期、富锦象屿金谷农产品有限公司120万吨玉米深加工等项目，实现泉林秸秆综合利用一期、年产5万吨高端铝型材等190个项目竣工投产。优化招商引资工作。突出长三角、珠三角、京津冀鲁闽等重点区域，开展专业化招商，扎实做好信息收集、甄别研判和跟踪洽谈工作，引进千万元以上项目72项，国内招商引资到位资金增长15%，实际利用外资增长10%。促进产业园区升级。完善各类园区基础设施，推进重点园区加快向生态、创新、特色、集约转型。支持各级园区盘活闲置土地，促进集约利用；鼓励发展“飞地经济”，引导新产业、新技术向园区集聚。推进市高新区创建国家级开发区，谋划建设综合保税区和秸秆高效利用循环经济园区。启动建设佳木斯国家级农业科技园区。

（二）深化结构调整，促进转型升级。坚持以增量带动结构优化，以创新促进产业升级。

在转变方式中发展现代农业。强化市场导向作用，创新产业化模式，实现农业提质增效，加快农业资源优势向经济发展优势转化。优化农业产业结构。适应农产品需求向品牌化、高端化、时尚化发展趋势，以绿色有机理念引领农业可持续发展，试行佳木斯优质水稻栽培技术规程地方标准，全力打造“百公里绿色稻米长廊”，建设百万亩优质水稻示范带。发展特色种养殖业，创新汤原亮子奔腾、桦南仙紫紫苏等企业的产业化模式，新增紫苏、杂粮、果蔬、大榛子、饲草等特色种植面积25万亩，栽培食用菌3.5亿袋；新建改扩建标准化规模养殖场20个，发展名特优水产养殖基地5个、稻田养鱼养蟹面积2万亩。推进“三减”行动计划，推广秸秆置换黄腐酸肥，完成测土配方施肥面积800万亩以上。加快生态高产标准农田建设，推进农田水利设施市场化运营。促进规模经营。完成土地确权登记工作，探索农村土地征收、经营性建设用地入市、宅基地制度改革有效途径，推动土地经营权有序流转，培育规范运行的新型农业经营主体100个，增强规范化管理水平和自我发展能力；吸引工商资本参与现代农业发展，鼓励合作社与工商资本嫁接联合、深度融合，提升产业层次和水平，实现从生产主体向全方位经营主体转变。支持金玛、金铭恒源等农业龙头企业扩大规模、创新品牌、创新标准，提升市场影响力。加快发展社会化服务体系，完善农业科技人员与新型经营主体对接包保机制，引导经营性主体发展专业化服务。支持辖区银行探索涉农信贷新产品，降低融资成本，扩大融资规模，基本实现应贷尽贷。创新营销模式。建立农产品质量安全可追溯体系，借助“互联网+农业”平台，充分挖掘定制经济潜力，推动绿色有机农产品全网直销，重点打响“佳木斯大米”和“佳木斯黑木耳”品牌，让优质农产品获得更大增值收益。

在做大增量中优化工业结构。把发展实体经济作为根本之策，推动供给侧结构性改革，提升工业产品竞争力。引导现有企业存量资源，通过联合、重组、改造、上市等方式，完善治理结构，创新商业模式，抓好品牌创建，提升技术创新能力，实现更好发展。开发适应市场需求的新产品，增加有效供给。重点推进中唯实业磨具冲压中心等投产项目尽快达产，润特科技与哈理工合作生产特种电缆等洽谈项目及早落地。贯彻《中国制造2025》规划意见，按照高端化、智能化、服务化方向，促进农机制造等主导产业，通过引进战略投资者、嫁接应用新技术等方式，优化产品结构，提高市场占有率，全力打造国家综合农机产业基地；积极发展绿色食品加工，建设绿色有机食品基地。注重绿色转型，加快培育节能环保、新能源、新材料等新兴产业，逐步形成工业多点支撑格局。认真落实企业减负优惠政策，打好降成本、补短板、增效益“组合拳”。增强县域经济实力，县域经济增长6.5%。深入实施非公经济发展三年行动计划和中小企业成长工程，新增小微企业3000户。

在消费升级中释放需求潜力。创新营销理念和方式，引入外部需求，激活域内消费，促进服务业跨界发展。做优商贸物流。加快佳木斯毅德商贸物流城等重点城市综合体项目建设，万达广场项目室外步行街投入运营；推进京东等国内大型第三方电商在我市建立分支机构，实现县级电商服务中心全覆盖；谋划建设佳通快递物流园区、综合物流园区和空港物流园，推动电商、快递、邮政和智能仓储联动发展。做实文化旅游。同江三江口生态旅游区、街津口赫哲旅游度假区创建国家4A旅游景区；以汤原大亮子河国家森林公园为重点，提档升级富锦国家湿地公园等18处旅游景区，开发航空旅游市场；启动文旅集团组建工作，整合文化和旅游资源，加强带有地域文化色彩的旅游产品和线路开发，加大线上线下推介力度，提供以游客为中心的便捷化服务，旅游业收入增长10%以上。做活现代金融。引进4家银行设立分支机构，筹建佳木斯农村商业银行；发展私募股权投资和风险投资基金，支持银行资本和社会资金组建村镇银行；扶持3户企业挂牌上市，借力资本市场寻求发展；搭建各类要素交易平台，开展融资租赁业务。从严打击各类非法集资、恶意逃废银行债务等行为，清理整顿投资理财公司，防范金融风险。培育会展保险、科技服务、商务咨询、文化创意、教育医疗等业态，推动服务业结构优化。

在不断创新中集聚发展动能。实施创新驱动发展战略，促进大众创业万众创新。发挥“互联网+”的先导作用，优化支持创业、创新、创客的环境。适应消费个性化、多样化趋势，引导中小微企业“专精特新”发展。加强创业孵化基地和政策、服务、培训体系建设，鼓励科技人员、大学生、农民、残疾人等各层面群体创新创业，推进大学生创业社区等创新创业基地规范化运行，充分发挥创业对拉动经济增长的乘数效应。注重新型商业模式、新经济现象、新技术变革的趋势性变化，强化企业创新主体地位和主导作用，对接高校院所开展深度合作，促进科技成果在我市转化落地，实现创新资源向企业聚集。新建产业技术创新战略联盟2个，新增国家高新技术企业5户、具有一定规模科技型企业20户，高新技术产业产值占工业产值比重超过35%。健全本地人才评价选拔体系，加大高技能人才培养引进力度；落实知识产权、科技成果转化为股权、期权等政策，鼓励各类人才依靠创新成果一夜致富。

（三）持续深化改革，激发市场活力。务实推进既具有年度特点又利于长远制度安排的改革举措，增强改革举措的针对性和有效性。

持续加大行政审批制度改革力度。动态调整权力清单、责任清单，规范重点审批项目的运行流程，建立中介服务清单，优化公共服务流程。全面推进不动产统一登记工作。继续深化公共资源交易管理体制改革。推进公共资源交易中心标准化、规范化、制度化建设，将所有经营性公共资源纳入中心公开配置。加快财政和投融资体制改革。完善预算体系，建立跨年度预算平衡机制，促进绩效管理结果应用；建立财政资金支付全程动态监控机制，保障预算执行严格规范高效；完善政府债务借用管还机制，积极推广应用PPP模式，创新融资渠道，防范债务风险。启动信访工作制度改革。拓宽信访途径，推进网上信访建设，畅通群众诉求渠道，健全矛盾纠纷多元化解机制；严格实行诉访分离，加大信访积案化解力度，引导群众在法治框架内解决矛盾纠纷，维护信访秩序。深化综合行政执法体制改革，解决交叉执法问题。加快“三权合一”的事业单位改革、商事制度改革及供销社、国有林场改革。

（四）实施外向拉动，拓展合作领域。强化互联互通，深化区域合作，积极融入国家“一带一路”和“龙江陆海丝路带”总体布局。

加快口岸通道建设。协调同江中俄铁路大桥俄方加快建设进程，力争江海联运大通道项目列入国家层面推动，支持黑瞎子岛开通陆路口岸，优化调整国际国内航线。提升对俄合作水平。强化佳富同抚对俄沿江开放协作，加强对俄跨境电子商务、跨境物流仓储、进出口商品加工、农业资源开发等方面合作。推进同江跨境经济合作区、万源通远东再生物资回收物流园等境内外园区建设，增强在俄投资企业配套功能。拓展对外开放领域。鼓励企业到境外设立营销网络，参与经营活动。集聚发展外向型产业，促进境外企业和本地产业对接融合。加强对俄、韩、日及港澳台地区的互访交流，吸引当地企业与我市开展经贸合作。积极开展与欧洲、新西兰等国家和地区的农牧业合作。实行“三个一”通关模式，提高进口产品落地加工、出口产品本地化率比重，全市外贸进出口额增长3%以上。

（五）坚持城乡统筹，提升人居品质。本着重基础、重民生、重生态、重品质的原则，系统性规划，高品质建设，规范化管理，打造秀美佳木斯。

推进城市总体规划修编，完善各类专项规划和控制性规划，加强“三区四线”管理，探索“多规合一”的具体实施办法。统筹重点，把握时序，完成长安西路等3条续建道路项目，建设胜利路和学府街立交桥，促进城区路网互联互通；推进中恒公司两炉两机等热源项目3项，新建改造供热管网35公里，推动热网连通和热源互调互补；加快佳东第二门站能源项目建设，新建改造燃气管网40公里，新增天然气居民用户1万户；实施地下综合管廊一期工程，建设供排水管网项目2项，完善城市功能。音达木河、王三五河在建工程全部完工，加强水源山公园、沿江公园等城市园林、沿江沿河景观升级改造及养护管理，启动柳树岛堤防环岛景观路项目，打造滨水城市。推进智慧城市建设，统筹实施智慧城管、智慧交通等信息化应用工程。推动东兴城小区与高新区的产城融合发展，塑造具有城市个性的建筑文化和空间特色。深入开展10项专项整治，健全城市管理督查问责机制，形成全民共治氛围。突出提升县城建设水平，完善镇村布局，建设美丽乡村100个。

（六）加快绿色转型，建设生态文明。落实绿色发展理念，推进绿色振兴，让良好生态成为可持续发展的竞争优势，惠及子孙后代。

强化大气污染防治。城区并网小锅炉供热面积80万平方米以上，推进锅炉除尘改造、堆场和工地扬尘污染治理，全面禁止使用劣质煤炭，完成年度燃煤小锅炉和运营黄标车淘汰任务，实行黄标车区域限行。开展水环境治理。完成江北水源地搬迁、东部城区污水处理场升级改造、西区调运污水、污泥处理等项目建设，启动四丰山、共和、向阳山等水库水污染治理和水质保护工程，力争完成英格吐河段整治任务。取缔自备井，总量控制地下水开发。加强生态环境保护。划定并严格执行生态红线保护规划，实施柳树岛生态修复建设，争取将我市湿地自然保护区纳入国家退耕还湿试点。创建国家级生态乡镇1个、省级生态县1个，省级生态村20个。全年完成造林9万亩，市区新增绿地8.5万平方米。推进工业污染源全面达标排放计划。治理农村面源污染，保护黑土资源。

（七）健全完善机制，开展脱贫攻坚。把扶贫开发作为重大政治任务，全面打响脱贫攻坚战役。

精准识别扶贫对象。认真开展贫困村、贫困户识别核实工作，建立公开公示和逐级审核制度，确保扶贫对象精准。明确脱贫责任目标。全力构建专项扶贫、行业扶贫、社会扶贫互为补充的大扶贫格局，逐年确定脱贫目标任务，2016年确保实现45个村、4万人脱贫。确定多元扶贫措施。着力破解基础设施瓶颈制约，以路、水、电、房为重点，改善贫困乡村基础条件。围绕产业脱贫、教育脱贫、就业创业脱贫、医疗保障和救助脱贫、社会保障兜底脱贫，因地制宜、因户施策，科学有效组织推进。强化政策服务支撑，加快跟踪对接，最大限度争取国省政策。层层签订包保责任状，以严格的督查问责，保障脱贫攻坚首战必胜。

（八）突出惠民利民，增进民生福祉。尽心竭力为基层群众解决急需办、能够办的实际问题，让百姓共享发展成果。

促进就业增长。创新小额担保贷款管理方式，城镇新增就业4.5万人（次），城镇登记失业率控制在4.4%以内。提高社会保障水平。新农合人均筹资标准提至560元；推行利用新农合基金为参保农民购买大病保险工作；落实城乡低保自然增长机制，将符合条件的贫困家庭纳入低保救助范围；完善贫困家庭医疗救助体系，全面开展临时救助工作。推进健康养老。市区新增公益性养老、社区日间照料床位1500张；鼓励社会资本投资养老产业，新建4所保障性医养融合养老机构；建设2处候鸟式养老项目，发展健康养老产业。改善人居条件。加快推进棚改五、六期项目，全面完成棚改年度任务，探索、落实多渠道安置棚改回迁户的新模式，有效减少商品房库存；改造农村泥（危）草房 9124户。实施城市既有建筑节能改造150万平方米，维修老旧小区楼房400栋；建立住宅小区电梯预警平台，规范住宅小区物业管理；依法解决住户多年不能回迁问题，化解信访积案；新建改建公厕26个、停车场15个；优化公交线路，增加运力投入，方便百姓出行。推进农村集中用水，提高城市供水质量。发展社会事业。促进义务教育均衡发展，完成32所农村薄弱学校改造，调整53所中小学教育网点布局；建立教师队伍补充机制，加强各级教师培训教育。促进职业教育专业设置与产业转型相适应。加强县级医院医联体建设，提高基层医院医疗水平；推动社会办医，满足差异化的健康需求；落实全面两孩生育政策。弘扬社会主义核心价值观，培育良好社会心态；打造艺术精品，实现文化惠民。发展以快乐舞步健身活动为主的群众体育运动。创新社会治理。制定社区工作准入目录，推进社区服务事项预约入户办理；新增规范化社区用房40个，创建省级“双百”示范社区9个。开展食品药品安全社会共治，争创国家级食品安全城市。严厉打击各类刑事犯罪，推进平安佳木斯建设。提升监管能力，严格监管责任，促进安全生产形势持续好转。健全防灾减灾救灾机制，提高应急管理水平。

四、推进政府管理创新

按照治理体系、治理能力现代化的要求，创新管理路径，提升服务效能。

（一）依法规范行政。落实《法治政府建设实施纲要（2015-2020）》要求，建立和完善法制政府指标体系，以法治思维、法治方式规范政府工作。依法设定权力、行使权力、制约权力、监督权力，推进政府决策和运行机制科学化、民主化、法治化。自觉接受人大法律监督、工作监督和政协民主监督，主动接受舆论监督、公众监督，强化效能监察和审计监督，习惯在受监督环境下工作、在法治轨道上用权。

（二）专业务实理政。创新发展理念，增强市场意识、规则意识、风险意识、发展环境意识，尊重规律，精准施策，全力稳定增长、优化结构、转换动力、防范风险，牢牢把握科学发展的主动权；创新工作方法，强化专业思维、专业素养，以改革创新的办法破解难题，以项目化管理的方式推动发展，以“踏石留印，抓铁有痕”的勇气和毅力抓好落实；创新日常管理，把“三严三实”专题教育成果落实到作风建设全过程，激发干事创业、担当作为的工作热情，严肃纠正懒政、怠政、为官不为等行为，提高政府执行力和公信力。

（三）全面从严治政。加强廉洁政府建设，严格履行“一岗双责”，自觉遵守廉政法规，知底线、守红线，着力在规范权力运行、打通联系服务群众“最后一米”等方面补缺、补弱、补短、补软，坚决查处影响制约科学发展、损害群众利益和影响经济发展环境的违纪违法案件，加快形成干事创业、风清气正的良好环境。

各位代表！新的目标催人奋进，新的征程任重道远。让我们在市委的坚强领导下，用恪尽职守诠释政府责任，用奋发有为回应各方期待，和衷共济，解放思想，勇于担当，真抓实干，为佳木斯更加幸福美好的明天而努力奋斗！
